# D-β-Hydroxybutyrate Is Protective in Mouse Models of Huntington's Disease

**DOI:** 10.1371/journal.pone.0024620

**Published:** 2011-09-12

**Authors:** Soyeon Lim, Adrianne S. Chesser, Jonathan C. Grima, Phillip M. Rappold, David Blum, Serge Przedborski, Kim Tieu

**Affiliations:** 1 Department of Neurology in the Center for Translational Neuromedicine, University of Rochester, Rochester, New York, United States of America; 2 Jean-Pierre Aubert Research Centre, Universite Lille-Nord de France, IMPRT, Lille, France; 3 Alzheimer and Tauopathies, Inserm UMR-U837, Lille, France; 4 CHRU, Lille, France; 5 Center for Motor Neuron Biology and Disease, Columbia University, New York, New York, United States of America; 6 Departments of Neurology, Pathology and Cell Biology, Columbia University, New York, New York, United States of America; Massachusetts General Hospital, United States of America

## Abstract

Abnormalities in mitochondrial function and epigenetic regulation are thought to be instrumental in Huntington's disease (HD), a fatal genetic disorder caused by an expanded polyglutamine track in the protein huntingtin. Given the lack of effective therapies for HD, we sought to assess the neuroprotective properties of the mitochondrial energizing ketone body, D-β-hydroxybutyrate (DβHB), in the 3-nitropropionic acid (3-NP) toxic and the R6/2 genetic model of HD. In mice treated with 3-NP, a complex II inhibitor, infusion of DβHB attenuates motor deficits, striatal lesions, and microgliosis in this model of toxin induced-striatal neurodegeneration. In transgenic R6/2 mice, infusion of DβHB extends life span, attenuates motor deficits, and prevents striatal histone deacetylation. In PC12 cells with inducible expression of mutant huntingtin protein, we further demonstrate that DβHB prevents histone deacetylation *via* a mechanism independent of its mitochondrial effects and independent of histone deacetylase inhibition. These pre-clinical findings suggest that by simultaneously targeting the mitochondrial and the epigenetic abnormalities associated with mutant huntingtin, DβHB may be a valuable therapeutic agent for HD.

## Introduction

Huntington's disease (HD) is a genetic neurological disorder caused by the expansion of a trinucleotide CAG repeat that encodes the polyglutamine region in the huntingtin protein. HD is characterized by a prominent loss of medium-size spiny neurons and the formation of protein aggregates in the striatum and the cerebral cortex [1].

Currently, the pathogenic mechanism leading to neurodegeneration in HD has not been fully elucidated. However, bioenergetic defects and epigenetic modifications have been proposed to be instrumental [2]–[4]. Metabolic impairment precedes the demise of striatal neurons [5], [6] and a reduction in mitochondrial respiration [7] have been detected in HD patients–consistent with the loss of complex II function induced by mutant huntingtin (mhtt) [8]–[10]. Furthermore, inhibition of complex II by malonate or 3-nitropropionic acid (3-NP) in animal models reproduces a pattern of striatal lesions similar to that seen in HD patients [2]. Numerous studies report that mhtt impairs mitochondrial function [11]–[13] and dynamics [14]–[16]. In addition to mitochondrial abnormalities, mhtt promotes epigenetic changes by binding to cAMP-responsive element-binding protein (CREB)-binding protein (CBP). When mhtt binds to CBP, the histone acetyltransferase activity [17] of CBP is inhibited, leading to global histone deacetylation [18]–[20] and consequently, gene suppression [3]. It is thus conceivable that mhtt can perturb neuronal function and survival by interfering with the activities of transcription factors. Consistent with this theory, treatment with various histone deacetylase (HDAC) inhibitors improves motor dysfunction, brain pathologies and life expectancy in animal models of HD [19]–[23].

D-β-hydroxybutyrate (DβHB) is a ketone body that has been demonstrated to be neuroprotective [24]–[28]. We have shown that in the 1-methyl-4-phenyl-1,2,3,6-tetrahydropyridine (MPTP) mouse model of Parkinson's disease, DβHB attenuates loss of dopaminergic neurons and functional deficits in a dose-dependent and stereospecific manner [28] by mitigating bioenergetic deficit. In light of that study, we hypothesized that DβHB may also be neuroprotective in HD. We report here that DβHB conferred neuroprotection in both a murine toxic model of striatal neuronal loss and a genetic model of HD. Importantly, in cell culture and animal models with mhtt expression, we uncovered a potential novel protective mechanism of DβHB: preventing the histone deacetylation induced by mhtt. We believe that both the bioenergetic and epigenetic actions of DβHB contribute to its neuroprotective effects in the HD models, and therefore, this molecule may be a novel therapy for this fatal neurodegenerative disorder.

## Results

### DβHB attenuates striatal lesions induced by 3-NP

**Figure 1:**
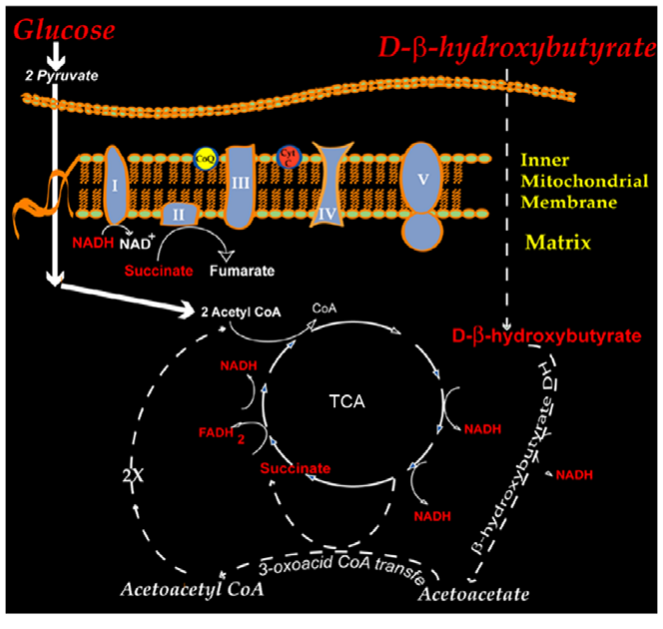
Metabolic pathways of DβHB in mitochondria. The metabolism of DβHB in mitochondria is stereospecific to the D isoform. Under normal physiological conditions, the level of DβHB is low but becomes dramatically elevated during starvation, increased fatty acid metabolism or pathological conditions such as diabetes. From its site of production in the liver, DβHB is released into the blood and circulated for utilization by other tissues. In general, the rate of ketone body usage in the brain is proportional to the concentration in the circulation [59]. Circulating DβHB readily crosses the blood-brain barrier and enters brain mitochondria where it is metabolized by mitochondrial β-hydroxybutyrate dehydrogenase to acetoacetate, which is subsequently converted to acetyl-coenzyme A to feed into the tricarboxylic acid cycle (TCA) cycle. The intermediate products generated from this cycle, NADH and succinate, in turn feed into the electron transport chain to subsequently generate ATP at complex V. Through this metabolic pathway, DβHB is an excellent alternative source of energy in the brain when glycolysis is not operative or when glucose supply is depleted such as during starvation [59].

**Figure 2:**
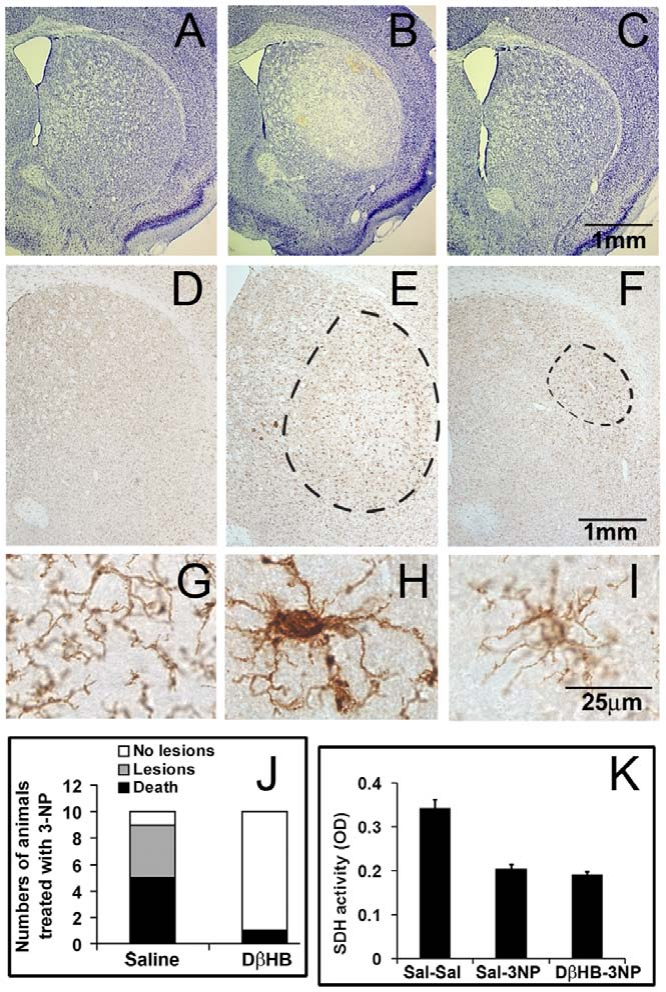
DβHB attenuates striatal lesions induced by 3-NP. Male C57Bl/6 mice (∼21 weeks-old) was infused subcutaneously (1 µl/h) with either DβHB (1.6 mmol/kg in saline) or vehicle (saline) using implanted osmotic minipumps. One day after surgery, each animal group was further subdivided into two groups to receive nine i.p. injections of either saline or 3-NP (50 mg/kg) at 12 h intervals. Five hours after the last 3-NP injection, animals were intracardially perfused with 4% paraformaldehyde. As compared to control (**A**), 3-NP induced significant cell loss (pale region) as seen here with Nissl stain (**B**). This lesion was not detectable in the group that received DβHB infusion (**C**). DβHB by itself did not affect the baseline level (not shown) of Nissl staining. Immunoreactivity for MAC-1/CD11b was observed after 3-NP treatment (**E**, **H**) in the lesioned area (outlined) as compared to the saline control group (**D**, **G**). Although 3-NP also induced discrete regions of increased microglia reaction in some animals that received DβHB (**F**, **I**), the immunoreactivity was not as dramatic as compared to the 3-NP treated group (**E**, **H**). **Panel J** summarizes the death rate and numbers of animals with striatal lesions induced by 3-NP in the groups that received either saline or DβHB infusion. **Panel K** demonstrates comparable magnitude of complex II inhibition in the two 3-NP treated groups, indicating that DβHB did not interfere with the function of complex II *per se*.

We previously demonstrated that DβHB conferred neuroprotection through the generation of succinate, a complex II substrate, in order to bypass complex I inhibition in the MPTP mouse model of PD [28]. In the present study, we hypothesized that in the neurotoxic model where 3-NP (a complex II inhibitor) was used to induce the striatal neuronal loss seen in HD, DβHB would similarly bypass this inhibition *via* its generation of the complex I substrate NADH (Figure 1). To this end, C57Bl/6 mice were injected with 3-NP in the presence or absence of continuous DβHB infusion using osmotic minipumps as previously described [28]. Consistent with its neurotoxic effect, in the group that received 3-NP alone (Figure 2B), Nissl staining of striatal sections revealed various sizes of bilateral lesions (marked pallor) in four of the five surviving animals (five deaths by the end of the 3-NP treatment, Figure 2J). In contrast, for the group that received pumps containing DβHB, none of the nine surviving animals (one death after the 3-NP treatment, Figure 2J) showed a detectable lesion (Figure 2C). In addition to striatal cell loss, 3-NP has been reported to induce gliosis [29]. Since DβHB prevented striatal cell loss, we determined whether this compound also had an effect on gliosis in these animals by assessing immunoreactivity for macrophage antigen complex 1 (MAC-1)/CD11b, a marker for microglia, in striatal sections. As seen in Figure 2E and H, microglial activity was dramatically upregulated by 3-NP in the areas corresponding to the lesion core. Discrete, darkly stained areas (circumscribed) were readily visualized from these sections. All five surviving animals from the saline group that received 3-NP showed strong microglial activation. Although microglial activation was observed in five out of nine surviving animals in the group that received DβHB (Figure 2F and I), microgliosis did not appear to be as robust as the saline group (Figure 2E and H).

To exclude the possibility that DβHB conferred protection by interfering with the levels of 3-NP in the brain, we performed histochemical analyses to compare succinate dehydrogenase (complex II) activity in striatal sections in animals treated with and without DβHB. Complex II inhibition induced by 3-NP was of comparable magnitude between these groups, demonstrating DβHB did not reduce the pharmacological effect of 3-NP (Figure 2K). Together, these results suggest that in this metabolic defect model of striatal neuronal damage, DβHB attenuates neurodegeneration probably through its well established bioenergic effect.

### DβHB attenuates motor deficits induced by 3-NP

**Figure 3:**
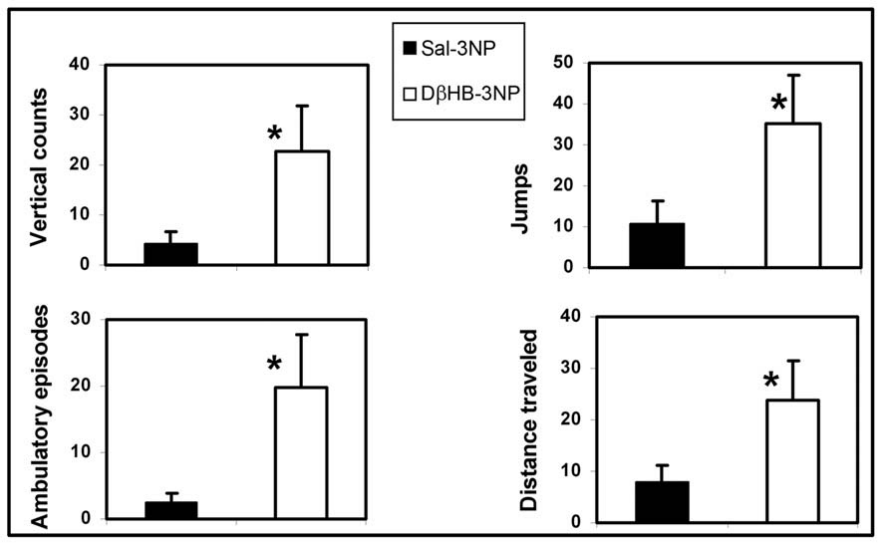
DβHB attenuates motor deficits induced by 3-NP. Male C57Bl/6 mice (∼21 weeks-old) were treated with 3-NP and DβHB as described in Figure 1. Five hours after the last 3-NP injection, mice were assessed for locomotor activities using infrared photobeam chambers. In the group that received saline infusion (*n* = 12), 3-NP injection induced dramatically impaired locomotor movements. In contrast, these abnormalities were attenuated in the 3-NP group that received DβHB infusion (*n* = 7). Units expressed as % of the saline injected control group (not shown) ± SEM. *p<0.05 as compared to the 3-NP treated mice that receive saline infusion.

In addition to the large striatal lesions observed, this 3-NP regimen induces severe locomotor abnormalities by the end of the treatment [29]. To quantify the magnitude of this behavioral abnormality in the presence of DβHB, C57Bl/6 mice were treated with 3-NP and DβHB as described in Figure 2 and five hours after the last 3-NP injection (the same time point used in Figure 2), locomotor activity was assessed using infrared photobeam chambers. As seen in Figure 3, animals treated with 3-NP alone were almost completely immobilized; however, DβHB partially improved motor dysfunction induced by 3-NP.

### DβHB delays motor deficits in R6/2 mice

**Figure 4:**
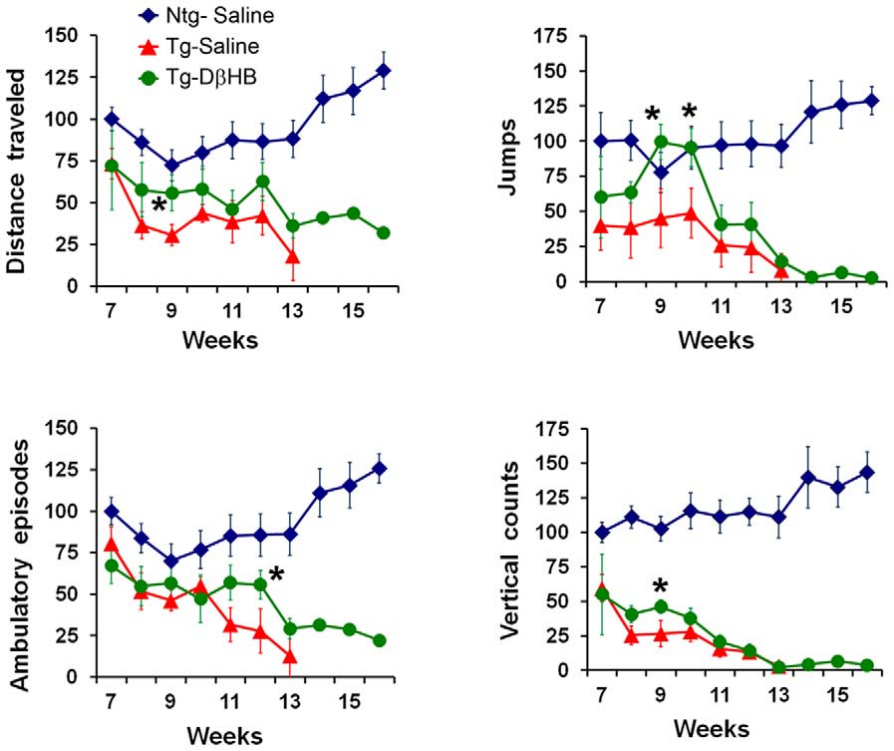
DβHB attenuates motor deficits in Transgenic-R6/2 mice. Six week old male transgenic (Tg)-R6/2 mice and non-transgenic (Ntg) littermates were infused with either saline vehicle or DβHB as described in Figure 2. Osmotic minipumps were replaced every two weeks for the entire study period. Locomotor activities were assessed using infrared photobeams chambers. Tg-R6/2 mice displayed significant impairment in locomotor movements. In contrast, these abnormalities were delayed and less severe in the Tg group treated with DβHB (*n* = 7). DβHB did not affect locomotor function of Ntg mice (not shown). Units expressed as % control of Ntg-Saline group at seven weeks old (*n* = 12). *p<0.05 as compared to the respective Tg-saline groups (*n* = 7) at the same time points.

Because HD is a genetic disorder, to assess the potential clinical relevance of DβHB for HD, we infused this compound in transgenic R6/2 mice, an HD model in which both mitochondrial dysfunction and epigenetic modifications have been demonstrated. Additionally, a clear and rapid progression of the disease (phenotype, neuropathology and life-span) in these mice make them an ideal model for us to test the protective effects of DβHB. Six week old male transgenic-R6/2 mice and their non-transgenic littermates were subcutaneously implanted with osmotic minipumps containing DβHB or saline for the entire period of study. Pumps were replaced every two weeks. Beginning one week after implantation, mice were assessed for locomotor activity using open field automated chambers. As seen in Figure 4, although moderate, DβHB significantly attenuated the severity of motor deficits in these animals. DβHB did not have an effect in non-transgenic wild type littermates (not shown).

### DβHB significantly extends life-span in R6/2 mice

**Figure 5:**
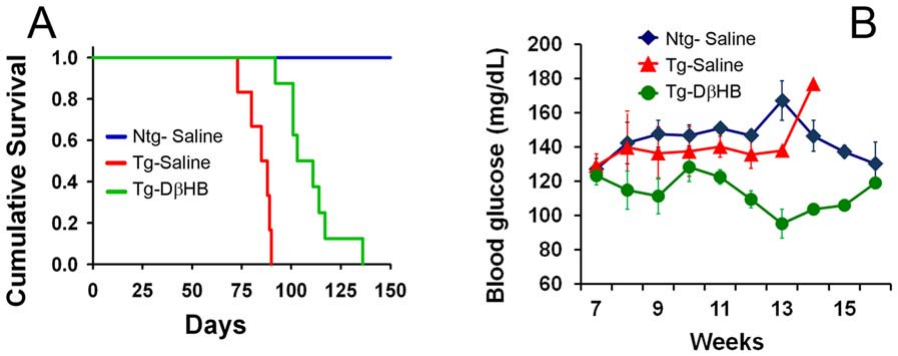
DβHB extends life expectancy and stabilizes glucose levels of Tg-R6/2 mice. Animals from Figure 4 were monitored twice daily for their survival rate (**A**). These mice were considered to reach their end stage if they were unable to right themselves after being placed on their back or died overnight. DβHB significantly extended the survival time in Tg-R6/2 mice. *n* = 12 (Ntg-saline), *n* = 7 (Tg-saline), *n* = 7 (Tg-DβHB). *p*<0.001 Kaplan-Meier analysis between Tg-sal and Tg- DβHB groups. These animals were also assessed weekly for glucose levels after six hours of fasting (**B**). One animal from the Tg-saline group survived up to 14 weeks and exhibited an elevated glucose level.

Life expectancy of the animals described in Figure 4 was monitored. In the group that received vehicle control, most transgenic animals died by 90 days. However, DβHB significantly extended the survival time (Figure 5A). The mean survival time in the DβHB treated group increased by ∼30.0% over the saline treated group (DβHB group = 108.7±4.3, saline group = 83.6±2.3, expressed as days ± SEM, *p*<0.001). None of the non-transgenic animals died by the end of this experiment. Perhaps due to the presence of polyQ aggregates in the pancreas leading to reduced mass and function of β-cell as well as interference with transcriptional regulation in islets, some of these transgenic mice have also been reported to develop diabetes towards the end stage of life expectancy [30]–[32]. To determine if this cohort of R6/2 mice also had this complication and whether DβHB was protective against this metabolic defect, blood glucose levels were monitored. We observed that up to the time point that most of these mutant mice died (13^th^ week), glucose levels remained normal, although for one mouse that survived up to 14 weeks, we did detect an elevated glucose level. Thus diabetes alone could not account for the increased death rate in these mice. In mutant mice treated with DβHB, glucose levels remained low for all animals (Figure 5B). However, it is unlikely that DβHB extended life expectancy in these transgenic mice through the improvement of diabetes because as discussed above, high blood glucose is not a prominent feature in this cohort. Regardless, because this metabolic disorder also occurs in HD patients [33], this stabilizing effect of DβHB on glucose levels may be a desirable feature for HD.

### DβHB prevents histone deacetylation induced by mhtt in R6/2 mice

**Figure 6:**
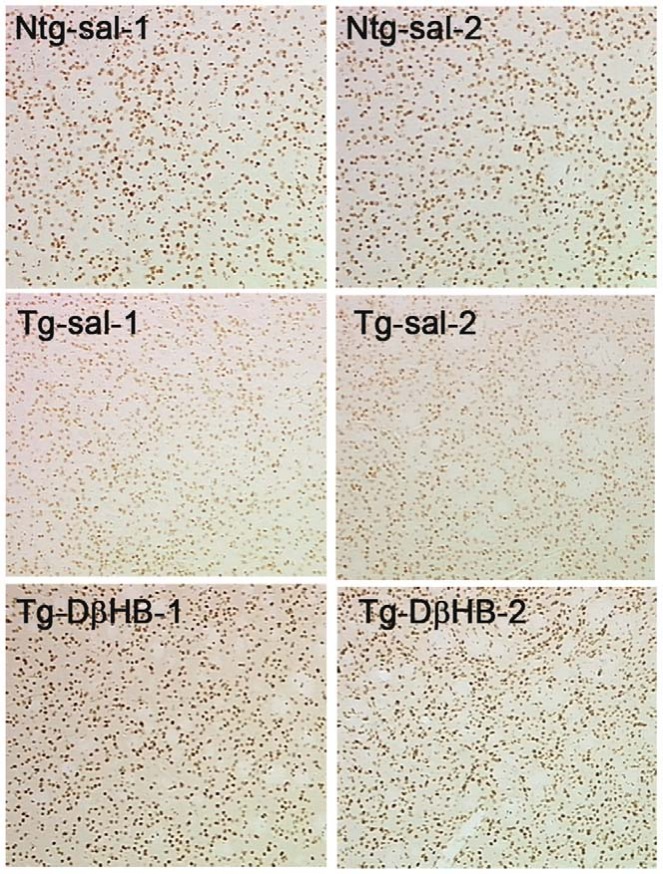
DβHB prevents histone deacetylation induced by mhtt. Animals recovered from the survival study were perfused and striatal sections were obtained and immunostained for histone H4 acetylation. Immunoreactivity was visualized by incubation in 3,3′-diaminobenzidine . There was less immunoreactivity of histone H4 acetylation in the transgenic animals as compared to the Ntg mice. DβHB treatment restored H4 acetylation in the Tg animals. *n* = 2 representative animals per group.

Neuropathology and gross morphological changes were also assessed in animals recovered from the experiments described in Figure 5. Three features commonly found in transgenic-R6/2 mice are striatal atrophy, protein aggregation and histone deacetylation. Although we observed a reduction in striatal volume in transgenic animals, we did not detect an overall change in striatal volume in these mutant animals treated with or without DβHB (data not shown). Nor did we detect an apparent difference in protein aggregation in striatal sections immunostained with an antibody (EM48, Chemicon International Inc.) against huntingtin (data not shown). However, DβHB dramatically restored the epigenetic abnormality in these animals. As illustrated in Fig. 6, consistent with other studies [20], [34], we confirmed histone deacetylation in transgenic mice by incubating striatal tissue sections with polyclonal antibodies against acetyl-histone H4. DβHB treatment blocked histone deacetylation in the transgenic mice. These results suggest that in addition to its well established bioenergetic effects, DβHB may also confer protection to the R6/2 mice through restoring epigenetic alterations induced by mhtt.

### DβHB prevents histone deacetylation induced by mhtt

**Figure 7:**
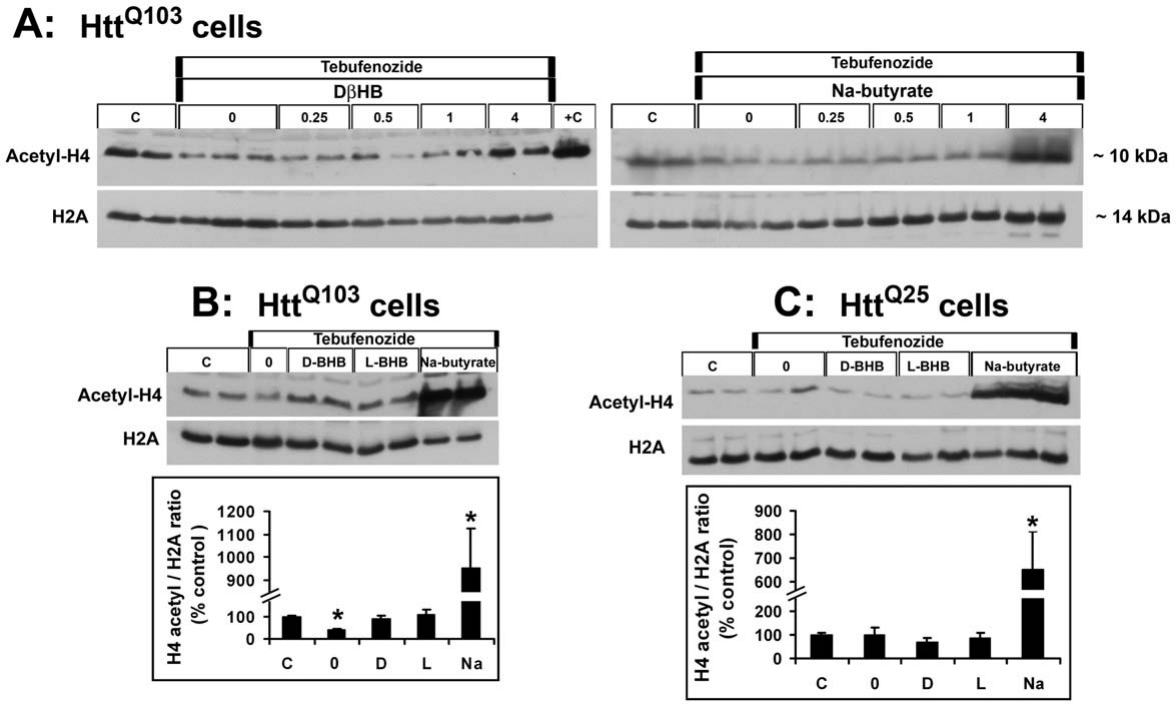
DβHB and LβHB prevent histone deacetylation induced by mhtt. (**Panel A**): Dose-response study of DβHB and sodium butyrate (0.25 mM-4 mM) in PC12 cells with inducible expression of Htt^Q103^ upon addition of tebufenozide (1 µM) using immunoblotting. Two microgram of histones extracted from Hela cells treated with 5 mM sodium butyrate (obtained from Upstate Biotechnology Inc.) was used as a positive control (“+C”). Levels of histone acetylation were quantified in cells with mutant (**Panel B**, Htt^Q103^) or normal (**Panel C**, Htt^Q25^) huntingtin 48 h after induction in the absence (“0”) or presence of equimolar concentrations (4 mM) of DβHB (“D”), LβHB (“L”) or sodium butyrate (“Na”) and compared with the non-tebufenozide treated control group (“C”). Data represent *n* = 6–10 per group (pooled from three separate experiments). *p<0.01 as compared to the control group (“C”) without tebufenozide treatment.

Primarily based on its similar chemical structure to the histone deacetylase (HDAC) inhibitor sodium butyrate, we hypothesized that DβHB would also attenuate histone deacetylation induced by mhtt *via* HDAC inhibition. To assess this possibility, we used stably transfected PC12 cells with inducible expression of human mutant (Htt^Q103^) and normal (Htt^Q25^) huntingtin. Htt^Q103^ cells have been reported to display histone deacetylation [18]. These stable cells were treated with or without 1 µM tebufenozide for the induction of huntingtin expression, in the presence or absence of DβHB, LβHB (a mitochondrially inactive isomer of DβHB) or sodium butyrate as the positive control. As illustrated in Figure 7, 48 h after induction, mhtt dramatically reduced the extent of acetylation on histone H4 (100%±3.12 vs 39.37%±6.71, Panels A and B) in the Htt^Q103^ cells. This reduction was absent in the Htt^Q25^ cells (Panel C), suggesting a specific effect of mhtt. The dose-response study shows DβHB and sodium butyrate significantly increased histone H4 acetylation at a concentration of 4 mM (Panel A) in Htt^Q103^cells. LβHB also had similar dose-response effect to DβHB (data not shown). Quantitatively, at an equimolar concentration (4 mM, Panel B), both DβHB and LβHB completely preserved the levels of histone acetylation (89.46%±11.66 and 105.25%±24.65, respectively). In contrast, sodium butyrate dramatically increased the acetylation level to 952.65%±24.65 – suggesting a different mechanism exists in these two classes of compounds. This pattern of dramatic difference was also detected in the Htt^Q25^ cells (Panel *C*) where DβHB and LβHB did not change the baseline of histone acetylation but a significant increase was induced by sodium butyrate.

### DβHB prevents histone deacetylation via an HDAC independent mechanism

**Figure 8:**
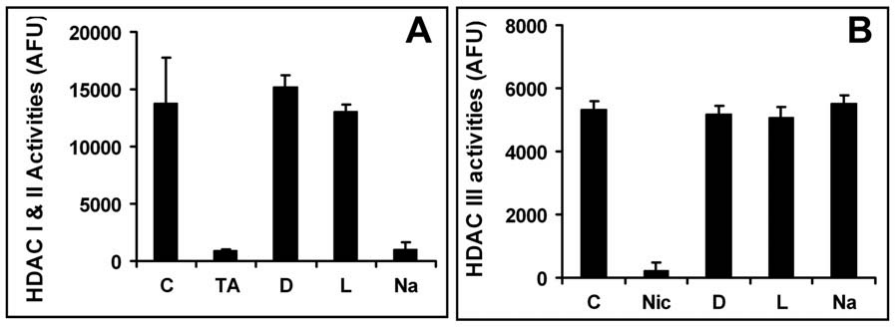
DβHB and LβHB do not inhibit HDAC activities. Hela nuclear extract was used as a source of class I and II HDAC activity (**Panel A**) where as recombinant human Sir1 was used as a source of class III HDAC activity (**Panel B**). HDAC activities were assessed in the absence (control, “C”) or presence of 4 mM DβHB (“D”), LβHB (“L”) or sodium butyrate (“Na”, an HDAC I and II inhibitor), 1 µM trichostatin A (“TA”, an HDAC I and II inhibitor) or 1 mM nicotinamide (“Nic”, an HDAC III inhibitor). Activities were quantified based on fluorescence intensity and expressed as arbitrary fluorescence unit (AFU). *n* = 6–16 per group from 5 independent experiments.

To further assess whether the effects of DβHB and LβHB on blocking histone deacetylation was a result of HDAC inhibition, we measured HDAC activities in the presence or absence of these compounds. Sodium butyrate served as a control. First, we measured the inhibitory effect of these compounds against classes I and II HDAC. Trichostatin A (1 µM) was used as an additional positive control for class I and II HDAC inhibition. As expected, both trichostatin A and sodium butyrate inhibited the activity of these enzymes (Figure 8A). However, DβHB and LβHB did not have this inhibitory effect. Even at a concentration up to 10 mM, these two compounds did not inhibit HDAC activity (data not shown). Since these compounds may potentially inhibit a third class of HDAC, we also assessed the effects of DβHB and LβHB on the enzymatic activity of HDAC class III. We also did not detect inhibitory effects of DβHB and LβHB against this enzyme (Figure 8B). Consistent with its role as an inhibitor of HDAC I and II, sodium butyrate also did not have an effect here. As a positive control, nicotinamide, an HDAC III inhibitor, almost completely inhibited this activity. Together, these results indicate that both mitochondrially active and inactive forms of beta-hydroxybutyrate prevent histone deactylation induced by mhtt via an HDAC-independent mechanism.

## Discussion

The neuroprotective effects of DβHB have been demonstrated in various models of neurological disorders [24]–[28]. We previously showed that in the MPTP mouse model of Parkinson's disease, DβHB attenuated loss of dopaminergic neurons and functional deficits in a dose-dependent and stereospecific manner *via* its bioenergetic effect [28]. In the present study, we report that DβHB is neuroprotective in both mitochondrial toxin induced striatal neurodegeneration and a genetic mouse model of HD. It is likely that the bioenergetic effects of DβHB contributed to the protection in these models. Although the epigenetic effects observed in the present genetic model may also play a role, it requires further studies.

In addition to its adverse effects on mitochondria, mhtt induces transcriptional dysregulation by binding to the cAMP-responsive element-binding protein (CREB)-binding protein (CBP), which also functions as a histone acetyltransferase (HAT) [17]. The expanded glutamine stretch in mhtt has been shown to bind to either the polyQ domain of CBP and sequester it in nuclear inclusions [35]–[37] or bind to the acetyltransferase domain (outside the polyQ domain) in CBP [18], [38], leading to the inhibiton of the HAT activity of CBP and ultimately, global histone deacetylation [18]–[20]. This reduction in histone acetylation, in turn, leads to gene silencing [3]. By increasing histone acetylation, histone deacetylase (HDAC) inhibitors improve motor dysfunction and brain pathologies, as well as increases life expectancy in animal models of HD [19]–[21]. Based on its similar chemical structure to the HDAC inhibitors sodium butyrate and phenylbutyrate, we initially hypothesized that DβHB would attenuate abnormal levels of histone deacetylation induced by mhtt *via* HDAC inhibition. Our subsequent studies demonstrated that although DβHB did prevent histone deacetylation induced by mhtt, the mechanism was not mediated by HDAC inhibition. Because DβHB is a well-established mitochondrial substrate, we assessed whether the observed epigenetic effect was mediated through mitochondria. The metabolism of DβHB to acetoacetate in mitochondria by β-hydroxybutyrate dehydrogenase is stereospecific [39]. Although cytosolic enzymes have been reported to metabolize LβHB in chicken liver [40] and sheep kidney [41], [42], there is no evidence to support the existence of either a mitochondrial L-3-hydroxybutyrate dehydrogenase or a racemase that interconverts the L- and D-isoforms of β-hydroxybutyrate [39]. Consistent with this, in our previous study using polarography we confirmed that DβHB, but not LβHB, could support mitochondrial respiration leading to higher ATP production [28]. Therefore, our data suggest that DβHB did not restore histone acetylation *via* a mitochondrial mechanism, because its mitochondrially inactive isomer, LβHB, produced the same epigenetic changes.

The precise mechanism by which DβHB prevents histone deacetylation induced by mhtt requires additional investigations. In general, there are a few potential mechanisms by which DβHB can increase histone acetylation in the presence of mhtt: 1) Inhibit HDAC activity, 2) Increase general histone acetyltransferase activity, 3) Reduce mhtt-induced protein aggregation, leading to less sequestration of CBP, and 4) Block the binding of mhtt to the active site of CBP, maintaining the normal HAT activity of this protein. We have eliminated the first possibility by demonstrating that DβHB did not inhibit any of the three classes of HDAC. Regarding the second possibility, we reasoned that if DβHB increased a general HAT activity, we would have detected an increase in histone acetylation when Htt^Q25^ cells were treated with this compound. This rationale is further supported by the fact that when these cells were treated with a general HDAC inhibitor (sodium butyrate), histone acetylation was increased. A general mechanism such as inhibiting HDAC or stimulating HAT should lead to an increase in histone acetylation, regardless of the presence of mhtt or normal htt. The fact that we only observed a DβHB-mediated increase in histone acetylation in cells with mhtt and that this increase was only to restore acetylation to the baseline level rather than the dramatic increase seen with sodium butyrate, led us to the remaining two possibilities. However, based on our immunohistochemical observations in both stable PC12 cells and R6/2 mice, DβHB does not appear to reduce protein aggregation (data not shown). Therefore, we are left with our last hypothesis. That is, this effect of DβHB is specific to mhtt and it may be mediated through blocking the binding of mhtt to CBP, leaving the HAT activity of this protein intact. It is worth noting that in addition to CBP, other proteins with HAT activity such as p300 and P/CAF may also interact with mhtt [18]. Thus, the effect of DβHB may not be restricted to just CBP.

Although DβHB extended the life span of R6/2 mice quite dramatically, DβHB did not produce a similarly striking improvement in motor deficits in these animals. This lack of robust effect may be a result of the experimental regimen. Because the half-life of DβHB is only about 1.64 h [43], it had to be delivered through osmotic minipumps. The large size of these pumps necessitated waiting until mice were six weeks old to implant them and begin treatment. However, R6/2 mice start to exhibit motor deficits as early as three-four weeks old and by six weeks already exhibit marked locomotor deficits [44], [45]. Thus, beginning DβHB infusion at six weeks was likely too late to get the maximum benefits of this molecule. Consistent with this theory, when these mice were treated beginning at seven weeks old, we did not detect such a clear trend of improvement in motor deficits (data not shown). As discussed in other reviews [46], [47], an early HD neurological phenotype, probably as a result of neuronal dysfunction, occurs before the overt appearance of protein aggregations or neurodegeneration. This neuronal dysfunction is likely mediated by interactions between mhtt and other proteins [47]. Relevant to our working hypothesis is the binding of mhtt to CBP, leading to histone hypoacetylation. The effects of gene silencing as a consequence of histone deacetylation very likely play a major role in early neurological deficits in HD patients and R6/2 mice. The ability of DβHB to prevent histone deacetylation induced by mhtt leads us to hypothesize that if given early enough and at a sufficient concentration to prevent the interactions between mhtt and CBP, DβHB may further attenuate neurological deficits in these animals. Future studies using mouse models with slower onset of symptoms or developing a means of delivering DβHB in mice younger than six weeks old are necessary to address this issue.

The short half-life of DβHB raises the question of whether this compound would be a feasible treatment for human diseases. We believe the first crucial step in drug discovery is to identify an active compound. Once such a compound has been identified, current pharmaceutical technology is readily available to improve such unfavorable pharmacokinetic profiles as a short half-life. For example, the compound of interest can be encapsulated in a polymer matrix facilitating slow release over time. This strategy is widely utilized by pharmaceutical companies and has been applied to control the release of levodopa (Sinemet® CR) for the treatment of Parkinson's disease. Relevant to the present study, orally active forms of DβHB with more favorable pharmacoketics are currently being developed by KetoCytonyx Inc, a biotechnology company dedicated to the use of ketone bodies for the treatment of various human diseases [27]. Similar, Accera Inc. has recently marketed Axona™, a medium chain triglyceride to generate ketone bodies, for the treatment of Alzheimer's disease. In addition to the pharmaceutical form of DβHB, non-pharmacological approaches such as a ketogenic diet (KD) and caloric restriction can also produce higher levels of this molecule in the body. Although not palatable and hyperlipidimic, KD has been used successfully for almost a century for the treatment of refractory epilepsy in children. KD has also been demonstrated to be beneficial in transgenic mouse models of Alzheimer's disease [48] and Amyotrophic Lateral Sclerosis [49] as well as in patients with Parkinson's disease [50] and Alzheimer's disease [51]. Caloric restriction (e.g., alternate day fasting) leading to higher serum DβHB concentrations is also neuroprotective [52]–[55].

In summary, the present study adds HD to a growing list of neurological disorders in which DβHB may confer neuroprotection. In addition to its well established mitochondrial effect, we may also unravel a novel epigenetic function of this molecule, leading to new insights into the mechanism by which it confers neuroprotection in treating epilepsy and other neurological disorders. Numerous studies have demonstrated the beneficial effects of increasing histone acetylation in HD [19]–[23]. Although we were unable to precisely elucidate the epigenetic mechanism of DβHB or to fully establish a direct link between its restoration of histone acetylation and neuroprotection, we believe the overall positive results in the present study serve as a starting point that encourages further investigations into the potential clinical relevance of this compound for HD.

## Materials and Methods

### Ethics Statement

All experiments were approved by the Institution Animal Care and Use Committee of the University of Rochester under the protocol number 2007-063.

### Animals and treatment

*3-NP model:* DβHB (1.6 mmol/kg/d in saline) or vehicle (saline) was infused (1 µl/h) in male C57Bl/6 mice (∼21 weeks-old) using subcutaneously implanted Alzet® osmotic minipumps (Model 2001, DURECT Corp., Cupertino, California, USA). This dosage of DβHB was chosen based on our previous dose-response study [28] showing that this concentration yielded approximately threefold increase in baseline plasma levels of DβHB (∼1.0 mM) and conferred maximal neuroprotection against MPTP toxicity. One day after implantation, each animal group was further subdivided into two groups to receive nine i.p. injections of either saline or 3-NP (50 mg/kg in 0.1 M PBS, pH 7.4) at 12 h intervals as described by Beal and colleagues [29]. Five hours following the last 3-NP injection, animals were anesthetized with pentobarbital (35 mg/kg, i.p.) and intracardially perfused with cold 4% (w/v) paraformaldehyde in 0.1 M PBS (pH 7.4). Their brains were removed, postfixed in the same fixative overnight at 4°C, cryoprotected in 30% (w/v) sucrose for 48 h at 4°C and then frozen in dry ice-chilled isopentane. Serial coronal sections (30 µm) spanning the entire striatum were collected free-floating in phosphate buffer prior to immunohistochemical analyses. *R6/2 model*: Female hemizygous R6/2 breeders (with ovarian transplant) were purchased from the Jackson Laboratories and bred with male B6CBAFI/J mice to maintain the same background. The offspring were genotyped using tail DNA. As with the 3-NP study, animals were subcutaneously infused with either saline vehicle or DβHB (1.6 mmol/kg/d) using Alzet® osmotic minipumps (Model 2002). Six week old animals were used because younger mice were too small to accommodate these osmotic pumps, which were replaced every two weeks throughout the study period. Only male mice were used.

### Locomotor activity testing

Automated chambers (Env-510, Med Associates, St. Albans, Vermont) were used to quantify locomotor activity. Each chamber (10.75″L×10.75″W×8″H), with sound isolation cubicles, is equipped with infrared photobeams (3 mm in diameter) separated by 24.4 mm on a horizontal plane, 39 mm from the floor of the chamber. A second set of photobeams located 57 mm above the horizontal photobeams divides the chamber vertically. Using the Open Field Activity Software® provided by the company, photobeam breaks were recorded every 5 minutes for 30 minutes to quantify vertical, traveled distance, jumps and ambulatory movements. For the 3-NP treated mice, animals were assessed five hours following the last 3-NP injection. R6/2 mice were assessed weekly, starting one week after the implantation of the pumps.

### Survival analysis

For the survival studies of R6/2 mice, we adopted the criteria described previously [20] to determine the end point of an animal. Animals were observed twice daily, morning and late afternoon. When the animals were inactive and unable to right themselves after being placed on their back, they were euthanized with a lethal dose of pentobarbital. Animals that died overnight were recorded the next morning. This study was done in the same groups of mice used for behavioral assessment.

### Assessment of striatal lesions in the 3-NP model

Striatal sections obtained from 3-NP injected mice were stained with thionin (Sigma-Aldrich) for Nissl substance to assess for the striatal lesions or immunostained for MAC-1/CD11b (Serotec) for microgliosis. Biotinylated secondary antibodies followed by avidin-biotin complex were used. Immunoreactivity was visualized using 3,3′-diaminobenzidine (DAB)/glucose oxidase/glucose.

### Blood glucose measurement

Glucose levels were measured in a drop of blood obtained from the mouse tails using a glucometer (AccuCheck Advantage®, Roche Diagnostics). After each session of locomotor activity testing, blood glucose levels were measured following six hours of fasting.

### Complex II histochemistry

As described previously [28], [56], animals were perfused with PBS containing 10% glycerol. Brains were rapidly removed and frozen in dry-ice cooled isopentane and stored at −80°C. Brains were sectioned at 20 µm throughout the entire striatum and mounted onto glass microscope slides at every 8^th^ section intervals. Complex II activity was revealed by incubating sections at 37°C for 20 min in 50 mM phosphate buffer (pH 7.6) containing 50 mM succinate as a substrate and 0.3 mM nitro blue tetrazolium (NBT) as an electron acceptor. Striatal optical density of NBT was digitalized and quantified by using Scion Image program (Scion Corp., Frederick, Maryland, USA).

### Cell cultures and treatment

The genetically engineered pheochromocytoma cells (PC12) were provided by Dr. Erik Schweitzer. The process of generating these cells is well described [57]. Briefly, these cells are stably transfected with a DNA insert encoding exon 1 of human huntingtin expressing 103 CAG (polyQ) repeats (Htt^Q103^) fused to EGFP. Control cells express the normal length of polyQ (Htt^Q25^). The expression of these proteins is inducible by ecdysone or its analog tebufenozide, which was provided by Dr. Fred Gage (Salk Institute, CA). These cells display abundant protein aggregates and cell death occurs by 48 h after induction of mhtt [57]. Cells were grown in T75 flasks in DMEM medium supplemented with 10% horse serum, 5% fetal bovine serum and 0.5 mg/ml G418. Two days later, spent medium was replaced with fresh medium with or without 1 µM tebufenozide for the induction of huntingtin expression, in the presence or absence of DβHB, LβHB or sodium butyrate. After 48 h of induction, cells were trypsinized and collected for histone extraction.

### Histone Extraction and Immunoblotting

Histones of cultured cells were extracted as described previously [58]. Cell pellets collected from T75 flasks as described above were Dounce homogenized in ice-cold lysis buffer (10 mM Tris-HCl, 50 mM sodium bisulfite, 1% Triton X-100, 10 mM MgCl_2_, 8.6% sucrose, pH 6.5). The nuclei were collected by centrifugation at 1,000× *g* for 10 min, washed three times with lysis buffer followed by one wash of 10 mM Tris-HCl, 13 mM EDTA. pH 7.4. The pellet was suspended in 0.1 ml of ice-cold H_2_O and concentrated H_2_S0_4_ was added to the suspension to give a final concentration of 0.4 N. After incubation at 4°C for at least 1 h, the suspension was centrifuged at 15,000× *g* for 5 min using a microcentrifuge. The supernatant was transferred and mixed with 1 ml of acetone. After overnight incubation at −20°C, the coagulated material was collected by microcentrifugation at 15,000× *g* for 5 min and air-dried. This acid soluble histone fraction was dissolved in H_2_O. Protein concentrations were determined using BCA assay (Pierce, Rockford, IL). Proteins (20 µg/lane) were separated by gradient 5–15% Tris/Tricine-PAGE, transferred to PVDF membranes and blotted with antibodies against acetyl-histone H4 (1∶2,000). Antibody against histone H2A was used for loading controls to indicate equivalent amounts of histones were loaded. All of the histone antibodies were purchased from Upstate Biotechnology Inc. (Lake Placid, NY). These primary antibodies were incubated overnight at 4°C. Secondary antibodies conjugated with horseradish peroxidase were used and visualized by chemiluminescence (SuperSignal Ultra, Pierce, Rockford, IL). Bands of interest were analyzed and quantified using Scion Image.

### HDAC Activities

Activities of HDAC classes I, II and III were measured using the fluorometric HDAC Assay Kits (Upstate Biotechnology Inc.) according to the instructions provided by the company. Briefly, Hela nuclear extract was used as a source of classes I and II HDAC activity. Recombinant human Sir1 served as a source of class III HDAC. Equimolar concentrations (4 mM) of our compounds of interest—DβHB, LβHB and sodium butyrate—were incubated with this extract and the signals were measured using a fluorescence 96-well plate reader (excitation = 365 nm, Emission = 450 nm). Trichostatin A (1 µM) was used as a positive control for class I and II HDAC inhibition whereas nicotinamide (1 mM) was used to inhibit class III HDAC. Signals were expressed as arbitrary fluorescent units (AFU) and were within the linear range of the standard curve.

### Statistics

All values were expressed as mean ± standard error of the mean (SEM). Differences between means were analyzed using either t-test, one-way or two-way analysis of variance (ANOVA), with the different types of mice, treatment or time as the independent factors. When ANOVA shows significant differences, Newman-Keuls post-hoc testing was used to test pair-wise comparisons between means. The null hypothesis was rejected at the level of 0.05. Survival data was analyzed using Kaplan-Meier analysis. All statistical analyses were performed using SigmaStat-3.0 (Jandel Corporation, San Rafael, CA).
